# Enhancing Psychological Well-Being Assessment Through Data Mining: A Case Study from Thailand

**DOI:** 10.3390/ejihpe15040061

**Published:** 2025-04-14

**Authors:** Asamaporn Treearpornwong, Thiyaporn Kantathanawat, Mai Charoentham, Paitoon Pimdee, Aukkapong Sukkamart

**Affiliations:** School of Industrial Education and Technology, King Mongkut’s Institute of Technology Ladkrabang (KMITL), Bangkok 10520, Thailand; asamaporn.189@gmail.com (A.T.); mai.ch@kmitl.ac.th (M.C.); paitoon.pi@kmitl.ac.th (P.P.); aukkapong.su@kmitl.ac.th (A.S.)

**Keywords:** data mining, mental health assessment, student mental health, Thailand, Waikato Environment for Knowledge Analysis

## Abstract

This study examines the psychological well-being (PWB) of lower secondary school students in Bangkok’s Secondary Educational Service Area Offices (SESAO) 1 and 2, using data mining techniques to analyze key influencing factors and develop a culturally adapted PWB questionnaire. The research framework is based on six components: autonomy, environmental mastery, personal growth, positive relationships, life purpose, and self-acceptance. Data were collected from 2543 students in the 2023 academic year and analyzed using the Waikato Environment for Knowledge Analysis (WEKA) program and the JRip rule-based classification model. Results indicate that personal growth is the most predictive in the classification performance of PWB, followed by positive relationships and life purpose. A newly developed PWB questionnaire was tested for reliability, with the Supplied Test Set (80:20) method yielding strong performance metrics, including accuracy (90.18%), precision (69.00%), recall (90.90%), and F-measure (78.40%). This study demonstrates data mining’s effectiveness in identifying factors influencing adolescent PWB within the Thai context. The findings provide educators and policymakers with insights for fostering student well-being and contribute to research by offering a validated, culturally relevant assessment tool.

## 1. Introduction

Digital technology’s development and use have transformed the world, removing traditional barriers of space and time and creating a globally connected, borderless society. This technological integration has facilitated the rapid exchange and advancement of knowledge, primarily through digital data, to streamline various tasks. Today, digital technology is embedded in every field and profession, allowing individuals worldwide to contribute to the generation and sharing of information, leading to the rise of big data. As a result, humans have created systems to store, process, and analyze this data for optimal use. Modern tools such as data mining, which combine statistics, artificial intelligence (AI), and databases, are essential in extracting valuable insights, patterns, and predictive models from vast datasets (Bharadiya, 2023). These tools are particularly useful in enhancing the efficiency of information retrieval systems (Gupta & Chandra, 2020). Moreover, data mining is being increasingly applied in various sectors, including Social Opinion Mining (Cortis & Davis, 2021) and educational data mining (EDM) (Elbourhamy et al., 2023; Luo, 2023), which have become integrated into everyday business and student lives. Adapting to these evolving technologies is essential for individuals in today’s rapidly globalizing world.

However, the increased prevalence of digital technology has also led to unintended negative consequences. Zohuri and Zadeh (2020) reported a concerning link between modern technology and the rising rates of youth suicide, with the increased use of smartphones, digital devices, and social media playing a significant role. Excessive engagement with texting and social media interactions has contributed to this troubling trend (Merino et al., 2024).

Adolescents, in particular, are vulnerable to the stresses and challenges of modern life, which, if not appropriately managed, can lead to mental health issues or psychiatric disorders. This problem is becoming more pronounced in Thailand, where rising rates of mental health concerns, especially among students, are being observed (Srijundaree et al., 2024). Adolescence is a critical phase characterized by significant emotional, physical, and social changes, which make young people particularly sensitive to the uncertainties of life. If these challenges are not addressed appropriately, they may result in long-term mental health problems (Thapar et al., 2012).

The situation in the United States offers a sobering perspective, as suicide has become a leading cause of death among individuals aged 10–24. In 2017, it was the second leading cause of death in this age group, and between 2007 and 2018, suicide rates among 14–19-year-olds increased by 56% (Zohuri & Zadeh, 2020). Globally, suicide ranks as the third leading cause of death among adolescents aged 12–19, behind accidents and homicides. According to the World Health Organization (2021), 720,000 individuals annually die by suicide, with suicide being the third leading cause of death among 15–29-year-olds.

With suicide among adolescents a growing concern worldwide, Chaniang et al. (2022) have also reported that it is the second leading cause of death among adolescents globally, which is consistent with statistics from Thailand revealing that suicide is the third leading cause of death among Thai adolescents (Pengpid & Peltzer, 2020).

The mental health of Thai children and adolescents continues to deteriorate due to stress, anxiety, and environmental pressures (Assana et al., 2017). This underscores the importance of careful monitoring by families, schools, and communities and implementing national policies to promote adolescent mental well-being. Adolescence, a time marked by physical, emotional, and hormonal changes, often brings with it feelings of anxiety, shyness, guilt, depression, and anger, making it a crucial period for mental health intervention. Early adolescence, transitioning from elementary to secondary school, is a vulnerable stage when young people face significant changes in their educational environment.

Additionally, a study in Italy by Tommasi et al. (2022) reveals that secondary school students experience lower academic well-being, self-esteem, and perceived teacher support than primary school students. Similarly, the 2021 Global School-based Student Health Survey conducted by the World Health Organization (2021) found that 17.6% of adolescents aged 13–17 in Thailand had experienced suicidal thoughts, with suicide being the fourth leading cause of death globally among those aged 15–19, and it accounts for 6% of all deaths in individuals aged 10–24.

Given the increasing mental health challenges faced by secondary school students (Tommasi et al., 2022), fostering psychological well-being (PWB) has become more important than ever. Adolescents undergo at least two major educational transitions: from elementary to lower secondary school and from upper secondary to higher education (Sundqvist et al., 2024). These transitions often involve adjustments to new environments, learning methods, and social contexts, requiring effective self-management. Promoting PWB is a key factor in achieving success, as good mental health enables individuals to recognize their accomplishments, fostering a sense of pride, self-worth, and overall life satisfaction (Prewnim et al., 2023).

Given these challenges, this research addresses the importance of promoting PWB in adolescents. Specifically, it aims to develop innovative assessment models using data mining to support PWB among Thai lower secondary school students.

Traditional psychometric validation techniques, such as factor analysis and item response theory (IRT), have been instrumental in refining psychological measures. However, recent advancements in data analytics suggest that computational methods, such as rule-based data mining, can provide new insights into psychological constructs (G. Chen et al., 2025). Unlike standard validation studies, which focus on ensuring internal consistency and construct validity, data mining approaches prioritize pattern recognition and classification, making them valuable for exploring multifaceted psychological phenomena such as well-being (Rodríguez-Conde et al., 2021).

Given the increasing complexity of adolescent mental health challenges, integrating data-driven techniques with traditional psychometric approaches can provide a more holistic understanding of well-being determinants. This study applies a novel rule-based data mining method to examine how different dimensions of the Ryff PWB scale interact in the context of Thai adolescents. By identifying the most influential factors contributing to adolescent well-being, this research aims to support the development of targeted interventions and personalized well-being assessments. The goal is to identify factors influencing these students’ well-being and develop an online PWB questionnaire (Roncancio-Moreno et al., 2025).

While the Ryff psychological well-being (PWB) scale has been widely used and validated in various populations, our study takes a different approach by leveraging data mining techniques to explore the most influential dimensions of PWB among Thai adolescents. Instead of revalidating the scale using conventional psychometric techniques, our study seeks to demonstrate how data-driven methods can complement traditional approaches in identifying key predictors of well-being. This novel application of data mining allows for a more nuanced understanding of adolescent PWB by uncovering underlying patterns that may not be as apparent in classical psychometric analyses.

Unlike previous research that applied data mining to predict academic achievement or assess self-regulated learning behavior, the present study focuses on adolescent psychological well-being (PWB) within a culturally specific Thai context. For example, Elbourhamy et al. (2023) utilized machine learning to forecast academic performance in a computer networking course using 11 different models in an interactive learning environment. Liu and Cocea (2017) explored theoretical frameworks for applying data mining to assess self-regulated learning behaviors.

In contrast, our study applies the JRip rule-based algorithm to classify and identify key psychological well-being factors among lower secondary school students in Bangkok. Beyond mere classification, the study aims to develop a reliable and culturally appropriate PWB assessment tool tailored for Thai adolescents. Our approach contributes to psychological assessment and practical interventions by empowering students, teachers, and families to understand and support adolescent well-being during a crucial developmental stage.

## 2. Literature Review

### 2.1. Understanding Psychological Well-Being in Adolescents

In adolescents, psychological well-being (PWB) is increasingly recognized as a multi-dimensional construct encompassing emotional, psychological, and social functioning (Keyes, 2002). Traditional assessments have focused on self-report surveys to measure flourishing versus languishing mental states. However, recent developments have emphasized the need for culturally sensitive and methodologically strong frameworks, particularly in non-Western contexts (Ryff & Keyes, 1995; Yi, 2012). Constructs such as “flourishing” have begun to take root in Asian educational and developmental paradigms (Whitaker et al., 2022), where mental health is often interwoven with family and academic performance (Schrader, 2023). 

As such, from a Thai study of the mental health situation of high school students aged 12–18 years during the COVID-19 outbreak in Bangkok, it was found that female students had a prevalence of depression ranging from mild to severe at 52.5 percent and a suicide risk of 29.2 percent. Factors associated with depression and suicide among high school students included being female, not living with parents, and having a history of treatment for psychiatric illnesses. Moreover, they have stress problems with studying, family, and friends (Thiamsaeng, 2023).

In an additional Thai study, data on the results from a mental health consultation service from a Thai Mental Health Hotline in fiscal year 2019 showed that adolescents aged 11–19 called in for consultation on high stress. The top 3 most common mental health problems were (1) stress and anxiety at 51.36%, (2) relationship problems at 21.39%, and (3) depression at 9.82%, respectively (Akkarasanakul et al., 2023). Therefore, preventing adolescent stress requires cooperation from various agencies, especially schools, which must monitor students’ stress, including promoting students’ knowledge and awareness of stress. To enable students to take proper care of themselves.

With the rise of big data in psychology (Harlow & Oswald, 2016), machine learning (Van Lissa, 2023), and computational psychology (Liu & Wang, 2022), new methods have emerged for classifying and predicting well-being states using behavioral and contextual variables. Ploke et al. (2024) demonstrated how survey-based machine learning models could effectively map flourishing and languishing states among adolescents, improving the precision of well-being categorization. These innovations underscore the importance of aligning psychological constructs with scalable digital tools to support adolescent development, especially in high-pressure academic and relational environments such as East Asia.

### 2.2. Socioeconomic and Cultural Determinants of Adolescent PWB

Family income sufficiency and cultural norms significantly influence adolescent well-being, often interacting in complex ways. Studies have confirmed a strong positive relationship between income adequacy and mental health outcomes (Khatri et al., 2024; Thavorn et al., 2018). However, recent work—including this study—extends these findings by demonstrating that financial stability gains added predictive power when paired with strong interpersonal relationships, especially with teachers. The combined effect of economic and relational factors is underexplored in existing literature, but this synergy may be particularly salient in collectivist contexts such as Thailand. Furthermore, Thai adolescents’ development is shaped by the tension between traditional values (such as family interdependence and Buddhist principles) and emerging societal pressures. Yi (2012) emphasizes that in East Asia, educational achievement is not isolated from psychological functioning; instead, both are deeply intertwined. This perspective is echoed by Fauzi and Kurniawan (2024) and Ashrafova (2024), who stress the need for culturally responsive education that aligns with family dynamics and respect for authority.

### 2.3. Role of Schools and Teacher Support

Beyond family structure, schools serve as central ecosystems for adolescent well-being (Huang & Chui, 2024). Teacher-student relationships are particularly influential: positive emotional support from teachers promotes academic motivation and protects against psychological distress (Pan et al., 2023; Zhu et al., 2022). These results align with Huppert (2014) and Y. Chen et al. (2024), who have argued that social environments—particularly those in schools—play a defining role in youth mental health. However, a gap remains in understanding how teacher support amplifies the benefits of other factors, such as financial security. In Thai and other East Asian settings, academic pressure coexists with expectations of respect and discipline, making the emotional quality of teacher interactions incredibly impactful.

### 2.4. Data Mining Applications in Adolescent Mental Health

Beyond theoretical discussions, data mining has seen increasing application in youth mental health research (McIsaac et al., 2021), offering concrete tools to classify and support at-risk students. Abdul Rahman et al. (2023) used machine learning to analyze behavioral health data from university students, achieving high accuracy in predicting psychological well-being levels. Their study illustrates how non-invasive, large-scale data collection can aid in developing personalized mental health interventions.

Similarly, Rochin Demong et al. (2023) built a personality trait-based recommendation model to improve students’ social well-being. Their use of supervised classification algorithms exemplifies how data-driven approaches can be tailored to psychological constructs relevant to adolescent populations. These applications offer methodological advancements and align with culturally embedded mental health constructs—especially in collectivist societies where social harmony and interdependence are paramount (Ashrafova, 2024; Fauzi & Kurniawan, 2024).

McIsaac et al. (2021) pioneered recursive partitioning through SIDES (Subgroup Identification Based on Differential Effect Search) to reveal social intersections impacting youth mental health outcomes in Canada. Analyzing over 21,000 responses from adolescents aged 11–15, they identified new gender-related social locations where mental health disparities emerged. This novel subgroup analysis supports intersectional approaches, showing how mental health experiences vary significantly based on overlapping social and demographic factors.

Luo (2021) proposed a mental health intelligence evaluation system integrating data mining with joint optimization algorithms. The system combines an improved decision tree with a modified artificial neural network (ANN) to reduce misclassification rates and enhance the accuracy of student mental health assessments. Simulation results demonstrated higher system stability and operational efficiency than traditional evaluation models, marking a step forward in intelligent, scalable school support systems.

Li (2021) investigated the psychological challenges of urban migrant children in China using a data mining model coupled with cloud computing. Survey data from middle and high school students revealed prevalent issues, including anxiety, withdrawal, and inferiority complexes. The study highlighted significant differences in social adaptability and emotional well-being between migrant and urban youth. This research showcases the potential of data-driven tools to address nuanced, context-specific disparities within vulnerable populations.

Hertz and Barrios (2021) examined the U.S. adolescent mental health crisis before and during COVID-19, drawing on national CDC Youth Risk Behavior Surveillance data. They argue for stronger school-community partnerships, emphasizing the urgency of collaborative strategies to mitigate the pandemic’s deepening impact on already fragile youth mental health. Although not a technical data mining study, their work reinforces the importance of institutional frameworks that can support data-informed early interventions.

Such work supports the notion that mental well-being is profoundly shaped by context and that digital technology, when responsibly employed, can offer new pathways for assessment, prediction, and early support in school and community settings.

### 2.5. Gaps in the Existing Literature

While considerable work has been completed on adolescent well-being, several critical gaps remain—especially in non-Western contexts. Much of the existing literature focuses on either mental illness or isolated well-being indicators, with less emphasis on holistic, culturally sensitive tools. Furthermore, few studies integrate empirical data mining approaches with culturally grounded frameworks, particularly in Thailand. There is a pressing need for research that identifies at-risk students through advanced analytics and incorporates cultural norms, socioeconomic realities, and local education systems. This section highlights the need for interdisciplinary studies that bridge psychology, education, and data science to develop practical mental health tools for youth.

## 3. Materials and Methods

### 3.1. Population and Sample

The population for this study consisted of 222,496 lower secondary school students within the Bangkok Metropolitan Region (BMR). The decision to sample approximately 100 students per grade level (Matthayom 1, 2, and 3) was based on methodological precedent and fieldwork feasibility. This sample size enabled meaningful statistical comparison between educational levels while keeping data collection practical across the selected Bangkok SESAO schools. A recent systematic review by Yağcı (2022) on educational data mining revealed that educational studies commonly utilize sample sizes ranging from 50 to over 9000 participants, with acceptable classification performance between 74 and 90% accuracy. This supports the adequacy of the current study’s design. The sample was selected through a two-step process:

#### 3.1.1. Simple Random Sampling of Schools

Ten schools were selected from SESAO Bangkok 1 and 2—five from each region. These two areas were purposefully chosen due to their demographic diversity, allowing the sample to reflect the educational heterogeneity of the broader urban student population.

#### 3.1.2. Stratified Random Sampling of Students

Within each selected school, students were stratified by grade level (Matthayom 1, 2, and 3), and approximately 100–120 students per level were randomly chosen, yielding a preliminary sample size of 3000 students.

Following data cleansing—which involved excluding incomplete, missing, or inconsistent responses—the final analyzable sample was 2543 students, equating to an 84.77% usable response rate. Surveys were administered from 1 February to 31 May 2024, with students completing the questionnaire on mobile devices within school settings, under supervision from teachers or guidance counselors. Each session lasted approximately 15–20 minutes, and participation was limited to students aged 13 or older.

### 3.2. Research Tools

The primary research tool was a questionnaire designed to assess the PWB of the students, based on Ryff’s scales of psychological well-being (SPWB), adapted to the Thai context. The Thai version of the Ryff psychological well-being scale was used to ensure contextual relevance, as validated by Thongthammarat (2022). The instrument demonstrated a total reliability of 0.901, with subscale coefficients ranging from 0.470 to 0.760. The scale’s concurrent validity was 0.510. A separate adolescent sample showed a Cronbach’s alpha of 0.80, further supporting its use in Thai youth populations.

The questionnaire was divided into two sections: Section 1, containing the demographic and background information (14 items), and Section 2, containing the psychological well-being, assessed through 18 items using a 6-point Likert scale.

The adapted version of Ryff’s scales of psychological well-being (SPWB) used in this study was validated for the Thai context. A pilot study was conducted with a sample of Thai adolescents, and the scale demonstrated strong internal consistency, with a Cronbach’s alpha of 0.80. Furthermore, previous studies have established its validity in Thailand (Akkarasanakul et al., 2023; Thiamsaeng, 2023; Thongthammarat, 2022) and East Asia (Yi, 2012). These findings confirm that the adapted SPWB is reliable for assessing PWB among Thai students.

#### Point Likert Scale Values

A 6-point Likert-type opinion scale assessed each student’s opinion (Taherdoost, 2019). The scale values were 1 (Strongly Disagree): 1.00–1.83, 2 (Disagree): 1.84–2.67, 3 (Somewhat Disagree): 2.68–3.50, 4 (Somewhat Agree): 3.51–4.33, 5 (Agree): 4.34–5.17, and 6 (Strongly Agree): 5.18–6.00. This 6-point scale differs from a traditional 5-point scale by not including a neutral midpoint, encouraging respondents to provide a definitive opinion (Taherdoost, 2019). It was specifically designed to measure students’ levels of agreement with statements related to their PWB.

### 3.3. Data Collection and Ethical Considerations

Data collection occurred on-site at schools, ensuring the environment was familiar to the students. The guidance counselors and homeroom teachers administered the questionnaire, taking care to screen students in line with ethical guidelines for research with human subjects. Only students aged 13 and above were eligible to participate, ensuring their informed consent and protecting their privacy.

Efforts were made to protect students’ confidentiality, particularly as some survey items touched on personal mental health. Despite these precautions, the survey experienced a minor non-response rate, primarily among students with mental health concerns, those with language barriers, and those apprehensive about privacy concerns related to personal data sharing. To reduce social desirability bias, students completed the questionnaire anonymously, with instructions emphasizing the confidentiality of responses. The presence of teachers was limited to logistical support, ensuring students did not feel pressured in their answers.

### 3.4. Handling Missing Data

As mentioned earlier, data cleansing involved the removal of incomplete responses, and the treatment of missing or erroneous data were handled through exclusion. No data imputation methods were necessary for this sample. A listwise deletion approach was used, removing entire responses with missing data. Given the low missingness rate (<5%), imputation techniques were deemed unnecessary, as their impact on overall results would be minimal.

### 3.5. Research Methodology’s Model Evaluation

The goal was to identify the best-performing model using multiple splitting techniques. After running the JRip classifier with various splits and cross-validations, performance metrics (such as accuracy, precision, recall, and F1-score) were analyzed to assess the model’s predictive power. The combination of rule-based classification (JRip) (Naseri et al., 2022) and multiple data-splitting methods ensured a thorough evaluation, enabling the selection of the most accurate model. The research analyzed data from the PWB questionnaire using data mining techniques (Kudyba, 2014) in the following steps:

#### 3.5.1. Data Cleaning

The collected data were checked for missing values and special characters and converted into the required format.

#### 3.5.2. Data Integration

Data from various sources (e.g., paper and digital formats) were combined into a unified dataset, and results were calculated based on the PWB criteria.

#### 3.5.3. Data Selection

Only complete responses were selected for analysis. The demographic characteristics of this study’s 2543 lower secondary students present a clear overview of the population’s composition (Table 1). In terms of gender, the majority of respondents were male (58.43%), with females comprising 40.74% and a small proportion identifying as “Other” (0.83%). The religious breakdown showed that most students followed Buddhism (93.12%), followed by Islam (5.19%), while Christianity and other faiths accounted for a minor percentage. The students were distributed across three grade levels: Secondary 1 (32.32%), Secondary 2 (35.82%), and Secondary 3 (31.85%).

Academic performance indicated that nearly half of the students (45.58%) had GPAs between 3.50 and 4.00. Moreover, most students came from the BMR (72.63%), while smaller proportions came from other Thai regions, such as the Central Region, with 14.83% of the respondents.

Parental income levels were mainly in the range of 100–199 baht per day (76.80%), and family income sufficiency was generally reported as adequate by 85.21% of the respondents. Additionally, most students reported good (44.75%) or excellent (39.56%) relationships with their family, friends, and teachers. Family status data revealed that most students came from married households (70.86%), with a smaller portion coming from divorced families or those who had lost a parent.

The demographic characteristics, particularly the high proportion of students from the BMR and the majority identifying as Buddhist, reflect the broader societal context of Thailand. Understanding these characteristics is crucial for designing interventions tailored to the unique needs of this population.

#### 3.5.4. Data Transformation

The raw data were transformed into a readable format and saved in CSV format, preparing for analysis in WEKA (Waikato Environment for Knowledge Analysis) and RapidMiner (Kotak & Modi, 2020).

### 3.6. Data Mining and Analysis Using JRip in WEKA and RapidMiner

Before the analysis, the raw data were cleaned and transformed. This involves handling missing values, normalizing or standardizing the data, and transforming it into a format suitable for machine learning algorithms. Proper preprocessing ensures the data is ready for analysis without biases or errors.

#### 3.6.1. Repeated Incremental Pruning to Produce Error Reduction (RIPPER) Algorithm

The JRip algorithm (a Java-based implementation of the Repeated Incremental Pruning to Produce Error Reduction, or RIPPER, algorithm) is a rule-based classifier (Alibo, 2018). Rule-based classifiers generate a set of “if-then” rules from the dataset, making predictions based on matching these rules. JRip is popular for its simplicity, ease of interpretation, and ability to handle large datasets with noisy data. This algorithm iteratively creates rules from the training data and prunes unnecessary ones to improve accuracy and avoid overfitting. JRip was chosen for its interpretability and efficiency in handling categorical data, making it well-suited for analyzing students’ psychological well-being scores. Unlike black-box models such as neural networks, rule-based classification allows for transparent decision-making, aligning with educational research standards (Allahyari et al., 2017).

#### 3.6.2. Waikato Environment for Knowledge Analysis (WEKA)

WEKA is a widely used machine learning software that provides several tools for data mining and model evaluation (Hussain et al., 2018). WEKA is a popular open-source software package developed by New Zealand’s University of Waikato in 1993. Initially released as a tool for agricultural applications, it became a general-purpose machine learning tool by 1997. It is especially powerful for rule-based algorithms such as JRip.

#### 3.6.3. RapidMiner

RapidMiner is another popular platform that offers a user-friendly interface for building machine-learning workflows and evaluating models. It was initially developed in 2001 by the Artificial Intelligence Unit of Dortmund University of Technology in Germany (Liermann, 2021). Initially named YALE (Yet Another Learning Environment), it was rebranded as RapidMiner in 2007. Similar to WEKA, it provides various machine-learning tools, including classification, clustering, and rule-based algorithms.

#### 3.6.4. Comparison of Tools: WEKA and RapidMiner

While it is true that WEKA and RapidMiner both implement the JRip algorithm, the rationale for using both platforms lie in exploring their unique strengths and how they influence the practical application of the algorithm. Specifically, our comparison aimed to evaluate differences in the following aspects:

User Interface and Ease of Use—WEKA and RapidMiner have distinct user interfaces that impact the ease of operation, particularly for users who may not have extensive experience with data mining tools. By assessing these platforms, we sought to identify which software provided a more intuitive workflow for preprocessing, executing, and interpreting JRip results.

Preprocessing Capabilities—Both tools offer varied preprocessing options, which can influence the performance of the JRip algorithm. For instance, RapidMiner has built-in features for data transformation and normalization that streamline preprocessing workflows. In contrast, WEKA’s functionalities often require users to employ external scripting or plugins for similar tasks. These differences in preprocessing flexibility may lead to variations in how efficiently the tools prepare data for analysis.

Computational Performance and Efficiency—The computational efficiency of the platforms can vary depending on how the JRip algorithm is implemented and executed. For example, WEKA is optimized for research settings, focusing on algorithm development and testing, whereas RapidMiner is designed with industry-level workflows in mind, prioritizing speed and scalability. By comparing both, we could identify differences in runtime, resource utilization, and handling of large datasets.

Output Interpretation and Reporting—The tools also differ in how they present results. RapidMiner provides a graphical interface for visualizing outputs, which can be helpful for non-technical stakeholders. WEKA, in contrast, offers more detailed text-based outputs that appeal to researchers needing granular insights into algorithmic behavior. Highlighting these distinctions provides practical guidance to future researchers and practitioners in selecting a platform for similar studies.

Reproducibility and Cross-Validation Processes—Both tools offer distinct implementations of cross-validation and data-splitting techniques, which may impact the reproducibility of results. For instance, RapidMiner’s drag-and-drop interface simplifies the configuration of workflows, potentially minimizing user error. Conversely, while flexible, WEKA’s scripting approach may require greater technical precision to ensure consistent results.

While WEKA and RapidMiner implement JRip, their differing user interfaces, preprocessing capabilities, and reporting tools offer distinct advantages. Both tools validated results across platforms, ensuring consistency in JRip model performance. While WEKA provided greater flexibility for algorithm tuning, RapidMiner’s visual workflow facilitated intuitive data exploration. Comparing outputs from both tools strengthened result reliability (Table 2).

#### 3.6.5. Algorithm Parametrization and Model Selection

Dol and Jawandhiya (2023) provide comprehensive overviews of various data mining approaches in educational research, including classification, clustering, and association rule mining. These methods serve distinct purposes: classification focuses on categorizing data into predefined groups, clustering identifies natural groupings within the data, and association rule mining discovers relationships between variables (Allahyari et al., 2017). Importantly, they emphasize the synergy between these strategies, where combining methods can yield richer insights.

In this study, the distinction between strategy and algorithm is crucial. The “strategy” refers to the overarching framework for model selection and evaluation, encompassing cross-validation, percentage splits, and test set approaches. In contrast, the “algorithm” pertains specifically to the JRip rule-based classifier used for classification. By adopting this distinction, the authors align with the established methodologies outlined by Dol and Jawandhiya (2023) while contextualizing the approach within the broader field of EDM.

### 3.7. Data Splitting Techniques

#### 3.7.1. K-Fold Cross-Validation

This method helps estimate the model’s performance, splitting the data into k subsets (folds). The model is trained on *k* − 1 folds and tested on the remaining folds. This process is repeated *k* times, ensuring each fold is in the test set once. The results are averaged to give a reliable estimate of model performance.

*k* values of 5, 15, and 20 were used in this study.

A higher value of *k* (e.g., 20) generally provides more stable results but has higher computational costs.

Cross-validation, especially with different values of *k* (i.e., 5, 15, and 20), is highly effective in ensuring that the model generalizes well to unseen data. Rotating through different portions of the data minimizes the risk of overfitting and provides a reliable estimate of the model’s accuracy.

Higher *k* values (e.g., 15 or 20) lead to more computationally expensive runs. However, they often result in more stable and accurate models because the training sets in each iteration are larger.

#### 3.7.2. Percentage Split

A classification model’s performance in machine learning is affected by many factors, such as the method of splitting the dataset and the type of machine learning technology used, with the level of accuracy dependent on the method used (Shujaaddeen et al., 2024). This study’s method splits the dataset by a specific percentage into training and testing sets. Common splits are:

- 80/20: 80% of the data is for training, 20% for testing.
- 66/33: 66% training, 33% testing.
- 20/80: 20% training, 80% testing.

A 20/80 split is rare and is generally not considered ideal for standard machine learning workflows due to the limited training data available in such a split. It might be used in specific edge cases, such as testing models in data-scarce environments or specific validation scenarios. (less common, but for edge cases). 80/20 and 66/33 are widely used and strike a reasonable balance between training and testing. The choice often depends on the dataset size, with larger datasets more forgiving than smaller test sets (e.g., 80/20). In comparison, smaller datasets may benefit from larger test sets (e.g., 66/33) to provide more robust evaluation metrics.

The percentage split allows a quick evaluation but may not generalize as well as cross-validation. Percentage splitting is a more straightforward method than cross-validation and can be helpful in quickly evaluating model performance. The 80/20 and 70/30 splits are standard and reasonably balanced training and testing. Unlike cross-validation, this method can be more prone to bias depending on how the data is split. A particular split might not fully represent the entire dataset’s distribution, potentially leading to over-optimistic or overly pessimistic results. Rao et al. (2008) have also observed that due to the increased computational power, it is now possible to calculate cross-validation performance on a much larger number of tuned models than possible.

#### 3.7.3. Supplied Test Set

In this approach, the test set is predefined. This study divided it into 80% training/20% test or 70% training/30% test. This allows the evaluation of a specific, unseen portion of the data.

The supplied test set approach is practical when there is a need to evaluate the model on a specific, separate portion of the data that was not used during training. This is useful in real-world scenarios where a model is deployed on new data. If the test set does not represent the dataset’s overall distribution, the model’s performance could be misleading.

Combining JRip rule-based classification, K-fold cross-validation, percentage splits, and a supplied test set is a valid and effective approach for analyzing and predicting outcomes from large datasets. Using multiple evaluation methods enhances the robustness of the model selection process, helping to ensure that the model generalizes well to unseen data. While JRip is a solid choice for interpretable rule-based classification, comparing its performance to other classification techniques (e.g., decision trees, support vector machines) may be worth seeing if a different algorithm performs better, especially for more complex datasets.

### 3.8. Pattern Evaluation

The model’s performance was evaluated based on key metrics: accuracy, precision, recall, and F-measure. Given potential class imbalances in PWB responses, precision and recall were analyzed using weighted F1 scores. If discrepancies arose between metrics, priority was given to recall to ensure all at-risk students were identified, as false negatives could underestimate mental health concerns.

### 3.9. Knowledge Representation

The model evaluation results were synthesized and described about the six components of PWB. These findings were used to develop an effective PWB questionnaire for future use with lower secondary school students in the Bangkok Secondary Educational Service Area.

While data mining methods, such as JRip rule-based classification and machine learning-based evaluation, provide powerful tools for uncovering hidden patterns in psychological data, they should be viewed as complementary rather than substitutive to standard psychometric techniques. Traditional psychometric approaches, such as factor analysis and reliability testing, remain essential for ensuring theoretical validity, construct measurement, and interpretability in psychological research. Researchers can enhance predictive accuracy by integrating data mining with established psychometric methods while maintaining the conceptual rigor necessary for psychological assessment. This hybrid approach allows a more comprehensive understanding of psychological well-being (PWB) by leveraging empirical data-driven insights and established theoretical frameworks.

## 4. Results—Part A: Characteristics of the Phenomenon

### 4.1. Psychological Well-Being (PWB)

Students’ psychological well-being (PWB) levels were categorized into three groups—good, fair, and poor—based on scoring criteria proposed by Kaya and Erdem (2021). Specifically, scores between 78 and 108 were classified as good, 48 and 77 as fair, and 18 and 47 as poor. The distribution of students across these categories revealed that a significant majority (67.72%) fell into the *Fair* group. Complete distribution statistics for the 2543 participants are provided in Table 3.

### 4.2. Questionnaire Results

Table 4 presents the descriptive statistics (means and standard deviations) for the 18 items across the six components of Ryff’s scales of psychological well-being (SPWB). The students rated personal growth as the most important contributor to their PWB (mean = 4.60, SD = 1.16), followed by positive relationships (mean = 4.06, SD = 1.23) and life purpose (mean = 4.01, SD = 1.35).

### 4.3. PWB Characteristics by Group (Good, Fair, Poor)

To investigate how contextual and interpersonal variables relate to students’ psychological well-being (PWB), respondents were divided into good, fair, and poor mental health groups based on their PWB scores. This section also details the outcomes of the rule-based classification model developed using the JRip algorithm, implemented in WEKA and RapidMiner. Among all tested configurations, WEKA’s supplied test set (80:20) method yielded the highest performance, confirming its effectiveness in building a reliable PWB classification model.

Model Performance (WEKA, Supplied Test Set 80:20):

- Accuracy: 90.18%
- Precision: 69.00%
- Recall: 90.90%
- F-measure: 78.40%

These metrics align with existing research emphasizing the value of robust evaluation protocols in mental health assessments (Elbourhamy et al., 2023; Liu & Cocea, 2017). A summary of all tested model configurations is shown in Table 5.

The final model generated 28 classification rules. These were synthesized thematically and mapped to the six PWB components across the three well-being categories.

### 4.4. Level of Mental Health

#### 4.4.1. Good Mental Health

Students in the good mental health category exhibited strong psychological resilience and overall well-being, supported by key environmental and relational factors:

Family Income Sufficiency: Most students in this group reported adequate financial support from their families, allowing them to focus on academics and daily needs without undue stress. This finding aligns with prior research highlighting the positive relationship between financial stability and psychological well-being (Khatri et al., 2024). Financial security helps mitigate stress, fostering academic and social thriving (Diener et al., 2018). Notably, this study identifies that family income sufficiency, combined with strong teacher-student relationships, is more influential in supporting student well-being (SWB) than merely achieving balance across studies, extracurriculars, and relationships (Douwes et al., 2023).

Family Relationships: Students in this group frequently described their family relationships as “excellent”. A nurturing home environment is critical for Thai adolescents’ positive psychological outcomes. This contrasts with findings from Pan et al. (2023), who analyzed 1968 Chinese EFL learners and found a positive correlation between PWB and academic engagement. The contrast highlights the importance of teachers cultivating supportive relationships that nurture students’ socio-emotional development.

Relationships with Friends and Teachers: Strong interpersonal connections with peers and educators also emerged as key contributors to student well-being (Campbell et al., 2022; Y. Chen et al., 2024; Pan et al., 2023; Tran et al., 2023). Supportive friendships and teacher-student interactions instill a sense of belonging, enhancing emotional resilience and social competence. These findings are consistent with Huppert (2014), who identified positive relationships as central to flourishing mental health. LaBelle (2023) further emphasized that social-emotional learning (SEL) programs can increase student resilience and promote mental well-being.

In summary, students in this category benefit from a robust, multi-layered support system—anchored by family, teachers, and peers—that nurtures emotional and psychological well-being.

#### 4.4.2. Moderate/Fair Level of PWB

The study’s most significant proportion of students fell into the moderate or fair mental health category. While these students generally function well, they often experience emotional stability and well-being fluctuations.

These students are not significantly impaired daily but may struggle to maintain consistent emotional balance. This observation aligns with Ryff and Keyes’ (1995) concept of individuals who are “getting by” rather than fully thriving.

Keyes (2002) differentiates between “languishing” and “flourishing” mental health, noting that individuals in the middle spectrum often express neutral or ambivalent feelings about their life satisfaction and interpersonal relationships.

These findings suggest the need for proactive, targeted interventions to strengthen students’ social support networks and address stressors such as academic pressure (Maymon & Hall, 2021), which could help elevate them to higher levels of psychological well-being.

#### 4.4.3. Low/Poor Level of PWB

Students in the *Poor Mental Health* group face more profound challenges, particularly in family relationships.

Family Relationships: Students in this group often reported strained or only “fair” relationships at home. Such family dynamics may serve as sources of stress rather than support, contributing to isolation and diminished self-esteem. Previous research shows that poor family relationships negatively affect self-acceptance and life purpose—two key dimensions of well-being. Campbell et al. (2022) found that UK students at higher risk of poor mental health often had histories of childhood trauma, identified as LGBTQ+, or were on the autism spectrum. Conversely, building strong social networks can buffer these risks.

Financial Strain: While financial instability was not the most frequently cited concern in this group, existing literature shows that economic hardship often worsens family tensions and psychological distress (Thavorn et al., 2018; Wilson et al., 2023). Students in economically challenged families may experience chronic anxiety, which impairs academic performance and social relationships.

These findings underscore the need for tailored support mechanisms such as family counseling, school-based mental health resources, and financial aid. According to Ryff (2021), early interventions that enhance family dynamics and promote social support can play a vital role in helping adolescents regain emotional balance and resilience (Hutten et al., 2022; Van Dierendonck et al., 2008).

## 5. Results—Part B: Assessment and Model Development

### 5.1. Data Analysis and Model Evaluation for PWB

The results from the classification models were synthesized in alignment with the six dimensions of PWB. The data analysis for promoting PWB utilized a model evaluation strategy and a rule-based classification algorithm (JRip) to compare performance metrics such as accuracy, precision, recall, and F-measure across platforms. The Supplied Test Set (80:20) method in WEKA exhibited superior classification performance, achieving 90.18% accuracy, 69.00% precision, 90.90% recall, and 78.40% F-measure. The student respondents were categorized into three PWB levels: good, fair, and poor.

### 5.2. Flow Chart of Study: Steps in Model Development

**Figure 1:**
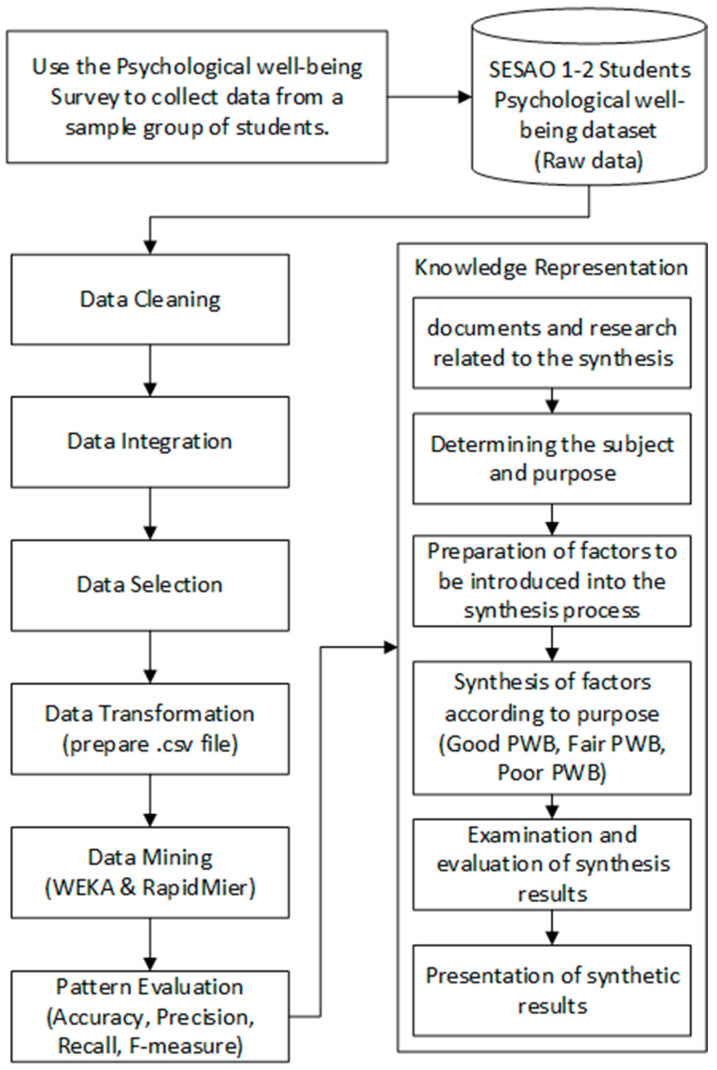
PWB development flowchart.

The model development for promoting mental health among lower secondary school students was developed using the model development framework with data mining techniques (Kudyba, 2014) and consisted of seven steps. Figure 1 illustrates the sequential steps in developing the classification model using data mining techniques.

### 5.3. Synthesized Classification Rules and Knowledge Representation

The model development for promoting mental health among lower secondary school students was developed using the model development framework with data mining techniques (Kudyba, 2014) and consisted of seven steps.

Data Cleaning: The data file collected from the mental health assessment tool is checked for data quality, such as missing values, memorable characters, and data conversion to the desired format.

Data Integration: Data from various sources is combined into a single format, such as integrating file types, paper-based data, and computing the mental health criteria results.

Data Selection: Data that respondents have thoroughly reviewed to ensure accuracy for all survey questions is selected.

Data Transformation: The data from the mental health survey is transformed into a readable and understandable format and converted into a CSV file to prepare for use with Vega (Waikato Environment for Knowledge Analysis: WEKA) software (version 3.8.6) and RapidMiner software (version 2024.0.1).

Data Mining: Complete data is used to search for models using Vega (WEKA) and RapidMiner software to compare accuracy values. This study uses the JRip algorithm for rule-based learning. The resulting model is represented as classification rules derived from training data, which is then tested with test data. Validation is performed using the K-fold cross-validation method, with k values set at 5, 15, and 20. The percentage split method is used with test data sizes set at 20%, 66%, and 80%. The supplied test set method is applied to split the data into 80% training and 20% test sets or 70% training and 30% test sets to identify the most suitable model for the data and desired outcomes.

Pattern Evaluation: The methods for creating predictive models are compared and selected based on performance evaluation criteria: (1) accuracy, (2) precision, (3) recall, and (4) F-measure.

Knowledge Representation: The results of the model evaluation are written as knowledge in the form of rules or conditions, with the following details:

1. Setting the Topic and Objectives: This step aims to develop a system for synthesizing relationships using data mining techniques for lower secondary school students. It involves identifying how different factors are integrated to form a model that helps the reader understand the data mining model and provides reasoning for its formation.
2. Preparing the Factors for Synthesis: The knowledge obtained from the data mining analysis in the form of rules or conditions is arranged into relationships, and the definitions of each factor affecting mental health, components of mental health, and guidelines for mental health care are reviewed. The results of the relationship synthesis are presented in written form.
3. Synthesizing Factors According to Objectives: The results from data mining analysis are justified, explaining why specific factors affect mental health and its components. The synthesized results are categorized according to mental health levels: good, fair, and poor.
4. Evaluating and Reviewing the Synthesis: The synthesized results are presented to the main and co-advisors for review and improvements based on feedback.
5. Presenting the Synthesis Results: The synthesized relationship results are compiled and presented in a table, such as Table 6: Components and their properties of poor, fair, and good mental health.

## 6. Discussion

### 6.1. Revisiting Objectives

This study was guided by five core objectives, each of which is revisited below in light of key findings.

The first objective—analyzing the demographics of the study population—was met through an in-depth examination of 2543 students, predominantly from the Bangkok Metropolitan Region and identifying as Buddhist (Table 1). These demographics provided vital context for interpreting psychological well-being (PWB) trends within a geographically and culturally distinct adolescent cohort.

The second objective involved classifying students into three distinct PWB levels using scoring criteria adapted from Kaya and Erdem (2021). Most students (67.72%) were categorized as “Fair”, reflecting findings from global adolescent well-being studies where moderate mental health states often prevail. Additionally, our findings showed that adolescents classified in the “Good PWB” group typically reported strong teacher and peer relationships (Y. Chen et al., 2024; Huang & Chui, 2024; Huppert, 2014; Pan et al., 2023; Zhu et al., 2022). These patterns underscore the need for nuanced, tiered interventions, particularly in transitional societies such as Thailand, where academic stress coexists with shifting cultural norms.

In the context of adolescent mental health research, data mining offers a valuable approach to uncovering hidden patterns within large-scale psychological data. By applying machine learning techniques such as rule-based classifiers, researchers can move beyond descriptive statistics to generate actionable insights, particularly in identifying at-risk youth based on multi-dimensional well-being factors (Yi, 2012). This approach enhances early detection and understanding of mental health vulnerabilities among adolescents, especially when traditional diagnostic tools fall short in culturally diverse or extensive educational settings. This study leverages data mining to enhance prediction accuracy and derive interpretable models that reflect the lived experiences of adolescents within specific cultural settings.

The third objective—developing a data-driven classification model—was achieved by implementing the JRip rule-based classifier in WEKA. The model demonstrated high accuracy (90.18%) and recall (90.90%) on the Supplied Test Set (80:20 split). These results affirm that transparent, interpretable models such as JRip can operationalize theoretical constructs such as Ryff’s dimensions (e.g., environmental mastery, positive relations) into concrete, if-then diagnostic rules. This is particularly advantageous in psychological research, where interpretability is often prioritized over black-box precision.

A particularly noteworthy and somewhat unexpected finding was the disproportionately high proportion of students (67.72%) classified within the “Fair” PWB group. While moderate well-being among adolescents is commonly reported in global literature, a deeper look into students’ item-level responses across the six Ryff dimensions reveals important nuances that may explain this central clustering:

Autonomy (AU): While some students demonstrated independent decision-making, many followed peers or prevailing social norms, especially in unfamiliar situations.

Environmental Mastery (EM): For example, in response to the item “*In general, I feel I am in charge of the situation in which I live*”, 52% selected “Somewhat Agree”. However, 41% also agreed that their daily roles often felt burdensome or overwhelming.

Personal Growth (PG): Responses to “*I gave up trying to make big improvements or changes in my life a long time ago*” showed a split, with over 42% choosing “Somewhat Disagree” or “Somewhat Agree”. This reflects a complex balance: while students aspired toward personal growth, rapid changes sometimes led to stress or anxiety.

Positive Relationships (PR): Nearly half of the respondents (47%) reported occasional difficulty maintaining relationships, signaling social strain or lack of consistent peer support.

Life Purpose (LP): Responses to the negatively framed statement “*I live life one day at a time and do not think about the future*” showed that 32% agreed at varying levels, suggesting uncertainty or lack of future-oriented motivation.

Self-Acceptance (SA): When asked about disappointment in personal achievements, over 59% showed agreement, hinting at underlying self-criticism or perceived underperformance.

In the Thai cultural context—where collectivist values, academic competition, and respect for authority are deeply embedded—such mixed patterns likely reflect latent psychological stress and adaptive social behaviors. Additionally, cultural norms around modesty and response moderation may have contributed to central tendency bias, where students avoid selecting extreme options. Thus, the “Fair” category may encompass students in psychological flux and those concealing distress beneath surface-level stability. These findings support the need for granular, culturally sensitive assessment tools and early-stage mental health interventions.

The fourth objective aimed to identify distinguishing characteristics across PWB categories. The extracted JRip rules revealed critical markers such as financial sufficiency, quality of peer and teacher relationships, and personal growth indicators. These factors resonated with Ryff’s framework and were grounded in contextually relevant adolescent experiences, reinforcing that well-being is not merely an internal state but a dynamic interplay between individual capacities and social ecosystems (Assana et al., 2017).

### 6.2. Implications for PWB Questionnaire Design

The study’s findings directly informed the design of a practically oriented, culturally sensitive PWB questionnaire tailored for Thai adolescents. In contrast to existing international instruments, this tool integrates:

Dimensions empirically validated as central to adolescent well-being in this context, such as financial sufficiency and teacher-student dynamics (Douwes et al., 2023).

Pattern recognition from JRip rules, ensuring alignment between statistical modeling and conceptual constructs.

High interpretability and applicability make the tool usable by school counselors, teachers, and local policymakers for diagnostic and preventative interventions.

The rule-based insights provide a foundation for adaptive screening, allowing practitioners to dynamically classify students without needing deep technical expertise—a crucial advantage for mental health work in resource-constrained settings.

### 6.3. Limitations and Future Directions

While the study presents significant contributions, several limitations must be acknowledged. Its focus on students in Bangkok may limit the generalizability of findings to rural or ethnically diverse areas. Moreover, reliance on self-report instruments raises concerns about social desirability and subjective bias.

Future work should expand sampling to include a broader demographic base, including rural and underrepresented ethnic communities, to enhance inclusiveness. Comparative evaluations using more complex classifiers such as Random Forest or XGBoost could also assess trade-offs between model interpretability and predictive power. Longitudinal designs are also recommended to track well-being changes over time, enabling evaluation of the long-term effectiveness of targeted school-based interventions.

## 7. Conclusions

This study successfully analyzed and designed a framework for promoting PWB among lower secondary school students in Bangkok’s SESAO 1 and 2. Using data mining techniques with WEKA and RapidMiner, the research applied classification and rule-based algorithms (JRip) to create models that predicted PWB levels. The Supplied Test Set (80:20) method in WEKA produced the highest classification accuracy at 90.18%, indicating that the model can effectively be used to develop a PWB questionnaire.

## Figures and Tables

**Table 1:** Respondents’ factors (*n* = 2543).

Items	Sample Size (*n* = 2543)	
	Number	%
Gender		
Male	1486	58.43
Female	1036	40.74
Other	21	0.83
Religion		
Buddhism	2368	93.12
Christianity	37	1.45
Islam	132	5.19
Hinduism	1	0.04
Irreligion	5	0.20
Secondary School Level		
Secondary 1	822	32.32
Secondary 2	911	35.82
Secondary 3	810	31.85
GPAX		
Less than 2.00	75	2.95
2.00–2.49	200	7.86
2.50–2.99	401	15.77
3.00–3.49	706	27.76
3.50–4.00	1159	45.58
Unknown	2	0.08
Domicile		
Bangkok Metropolitan Region	1847	72.63
Central	377	14.83
Western	8	0.31
Eastern	24	0.94
Northeastern	191	7.51
Northern	58	2.28
Southern	38	1.49
Income from parents per day (baht)		
Less than 100 baht	346	13.61
100–199 baht	1953	76.80
200–299 baht	215	8.45
More than 300 baht	29	1.14
Relationships with Family		
Excellent	1006	39.56
Good	1138	44.75
Fair	352	13.84
Poor	45	1.77
Bad	2	0.08
Relationships with Friends		
Excellent	1176	46.24
Good	1133	44.55
Fair	203	7.98
Poor	31	1.22
Bad	-	-
Relationships with Teachers		
Excellent	564	22.18
Good	1446	56.86
Fair	495	19.47
Poor	38	1.49
Bad	-	-
Family status		
Marriage	1802	70.86
Divorced	611	24.03
Father or Mother Deceased	115	4.52
Relatives	15	0.59
Average Family Income per month (baht)		
Less than 10,000 baht	213	8.38
10,000–30,000 baht	1343	52.81
30,001–50,000 baht	552	21.71
More than 50,000 baht	435	17.11
Sufficiency of Family Income per month		
Adequate	2167	85.21
Inadequate	376	14.79

**Table 2:** WEKA and RapidMiner comparisons.

Feature	WEKA	RapidMiner
Interface	Text-based, research-focused	Visual GUI, industry-friendly
Preprocessing	Requires external scripting/plugins	Built-in transformation tools
Performance	Research-optimized	Scalable for large datasets
Output	Detailed, text-rich	Visual, presentation-ready
Reproducibility	Manual precision required	Workflow drag-and-drop

**Table 3:** PWB levels (*n* = 2543).

PWB Level	Population (*n* = 2543)	
	Number	%
Good Mental Health	803	31.58
Fair Mental Health	1722	67.72
Poor Mental Health	18	0.71

**Table 4:** Ryff’s SPWB item means and SDs.

Items	Item	Mean	SD
Autonomy (AU)	AU	3.86	1.11
(1) People with strong opinions influence me.	AU1	3.49	1.14
(7) I have confidence in my opinions, even if they differ from those of most others.	AU2	3.70	1.10
(13) I judge myself by what I think is important, not by the values of what others think is important.	AU3	4.40	1.10
Environmental Mastery (EM)		3.86	1.07
(2) In general, I am in charge of the situation in which I live.	EM1	4.11	0.91
(8) The demands of everyday life often get me down.	EM2	3.24	1.27
(14) I am good at managing the responsibilities of daily life.	EM3	4.24	1.03
Personal Growth (PG)		4.60	1.16
(3) Having new experiences that challenge how I think about myself and the world is important.	PG1	4.84	0.98
(9) For me, life has been a continuous learning, changing, and growth process.	PG2	4.83	1.00
(15) I gave up trying to make big improvements or changes in my life long ago.	PG3	4.13	1.51
Positive Relationships (PR) with others	PR	4.06	1.23
(4) Maintaining close relationships has been challenging and frustrating for me.	PR1	3.67	1.30
(10) People would describe me as giving and willing to share my time with others.	PR2	4.28	1.01
(16) I have not experienced many warm and trusting relationships with others.	PR3	4.24	1.39
Life Purpose (LP)	LP	4.01	1.35
(5) I live one day at a time and do not think about the future.	LP1	4.28	1.46
(11) Some people wander through life, but I am not one of them.	LP	4.16	1.29
(17) I sometimes feel as if I have done all there is to do in life.	LP3	3.59	1.29
Self-acceptance (SA)	SA	3.85	1.24
(6) When I look at the story of my life, I am pleased with how things have turned out so far.	SA1	4.10	1.20
(12) I like most parts of my personality.	SA2	4.11	1.12
(18) In many ways I feel disappointed about my achievements in life.	SA3	3.35	1.41

Note: Q1 = Questionnaire item 1. Sources: Cortés-Rodríguez et al. (2023), Klainin-Yobas et al. (2020), Manchiraju (2020), Ryff and Keyes (1995), Ryff and Singer (2006), and Ryff (2021).

**Table 5:** Model performance metrics using data mining techniques.

Test Options	Accuracy (%)	Precision (%)	Recall (%)	F-Measure (%)
Weka Analysis				
K-fold Cross-validation (5 folds)	82.89	75.10	74.30	74.70
K-fold Cross-validation (10 folds)	82.58	74.20	73.50	73.80
K-fold Cross-validation (20 folds)	82.82	73.50	75.60	74.50
Percentage split: 20%	80.19	72.50	66.40	69.30
Percentage split: 66%	83.24	72.40	79.90	76.00
Percentage split: 80%	82.91	78.20	74.30	76.20
Supplied Test Set (70:30)	87.74	72.20	89.70	80.00
Supplied Test Set (80:20)	90.18	69.00	90.90	78.40
Rapid Miner Analysis				
K-fold Cross-validation (5 folds)	81.95	60.52	53.11	56.57
K-fold Cross-validation (10 folds)	81.56	58.11	56.48	57.28
K-fold Cross-validation (20 folds)	81.32	54.97	53.12	54.03
Percentage split: 20%	67.75	22.58	33.33	26.92
Percentage split: 66%	81.83	53.90	51.03	52.43
Percentage split: 80%	80.35	51.90	51.42	51.66
Supplied test set (70:30)	81.13	61.29	57.88	59.54
Supplied test set (80:20)	80.35	51.90	51.42	51.66

**Table 6:** Components and their properties of poor, fair, and good mental health.

	Bad PWB	Fair PWB	Good PWB
Autonomy	Students have low self-confidence in their opinions, rarely express or reveal their needs, and listen to others more. Students are very concerned with social pressures or trends in thinking or acting and care more about those around them than themselves.	Students have confidence in their own opinions and can sometimes make decisions independently. They can occasionally choose what is best for themselves and exhibit self-confidence in certain situations. However, they follow societal trends when making decisions in unfamiliar circumstances.	Students confidently form their viewpoints and make autonomous decisions based on what best suits their needs. They are capable of choosing without succumbing to external pressure or influence. These students show resilience against societal expectations and can manage their actions independently. They assess themselves according to personal values and internal standards.
Environmental Mastery	It is difficult for students to adapt to situations and environments or manage multiple daily responsibilities.	Students can manage various situations, even though the outcomes may not always be as desired. Additionally, they can handle their daily responsibilities effectively.	Students effectively manage daily responsibilities and handle various situations to meet their needs. They see their roles as students, children, or friends as positive. Additionally, they are confident in managing their surroundings, organizing daily life, utilizing opportunities, and shaping environments to align with their values and needs.
Personal Growth	Students feel that encountering new experiences does not challenge their self-perception and worldview, and sometimes, students feel that past and present experiences do not result in changes in their lives.	Students are relatively satisfied with their appearance and personality and are interested in self-development. However, rapid changes can cause them stress or anxiety.	Students love and are satisfied with their appearance and image. They are constantly striving to change and develop themselves. Moreover, students have a positive attitude towards change, which makes students grow both physically and mentally.
Positive Relationships	The outstanding characteristics of the students are: maintaining relationships with others is not difficult. Students can get along well with others, have love and good friendships with others, including seeing the characteristics of relationships with others positively, understanding giving and receiving, having trust, and being able to give love and forgive others.	Sometimes, maintaining relationships with others can be difficult for students. With time to get to know others, they are willing to open up, socialize, and form good friendships.	Students have positive relationships. Maintaining relationships with others is not difficult. Students can get along well with others, understand giving and receiving, and have trust, love, and good friendships.
Life Purpose	Students who lack clear life goals often experience feelings of unfulfillment, which can lead to a loss of meaning and direction in their lives. When they lose the beliefs that give them a sense of purpose, they struggle to find significance in past experiences and may feel disconnected from the potential meaning in their current life.	Students are flexible in setting life goals and place importance on being able to make independent decisions in some matters.	Students have important goals, motivating them to develop themselves and giving meaning to their lives. Students also value their own decisions and are committed to achieving their goals.
Self-Acceptance	Students are dissatisfied with themselves in the past and present and do not like their personality characteristics, such as their appearance, characteristics, and habits, which can easily cause anxiety and stress when something goes wrong with them. The student’s outstanding characteristic is that the student is happy with his/her past success. This is a good starting point for self-acceptance and having a good attitude towards himself/herself in the past and present.	Students are generally satisfied with themselves and recognize their self-worth, with rare occasions of disappointment in their achievements.	Students have a positive attitude towards themselves and have confidence and self-worth while accepting both good and bad aspects of themselves. They also like to seek new experiences that challenge their self-perception and worldview and feel optimistic about their past life.

## Data Availability

The datasets used and/or analyzed during the current study are available from the corresponding author upon reasonable request.

## Abbreviations

The following abbreviations are used in this manuscript:

AI	artificial intelligence
BMR	Bangkok Metropolitan Region
EDM	educational data mining
JRip	rule-based algorithms
PWB	psychological well-being
RIPPER	Repeated Incremental Pruning to Produce Error Reduction Algorithm
SESAO	Secondary Educational Service Area Offices
SPWB	scales of psychological well-being
WEKA	Waikato Environment for Knowledge Analysis program
WHO	World Health Organization
